# Supplementary material for: Discovery and pharmacophoric characterization of chemokine network inhibitors using phage-display, saturation mutagenesis and computational modelling
Source: Nat Commun. 2023 Sep 16;14:5763. doi: 10.1038/s41467-023-41488-z (PMC10505172; doi:10.1038/s41467-023-41488-z)
Supplement: Supplementary file 4 — Source Data [file 41467_2023_41488_MOESM4_ESM.zip › SourceData/scripts/xattune_20230807170606.tar!/xattune/analysis/attune_ic50/SV_E6_CCL5_EVA4_plot.pdf]

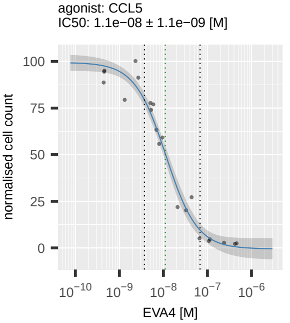
agonist: CCL5
IC50: 1.1e−08 ± 1.1e−09 [M]
normalised cell count
100
75
50
25
0
10^{-10}
10^{-9}
10^{-8}
10^{-7}
10^{-6}
EVA4 [M]
